# Highly Stable Persistent Photoconductivity with Suspended Graphene Nanoribbons

**DOI:** 10.1038/s41598-018-30278-z

**Published:** 2018-08-07

**Authors:** Hiroo Suzuki, Noritada Ogura, Toshiro Kaneko, Toshiaki Kato

**Affiliations:** 1 Department of Electronic Engineering, Tohoku University, Aoba 6-6-05, Aramaki, Aoba-ku, Sendai 980-8579 Japan; 2 Japan Science and Technology Agency (JST)-PRESTO, Aoba 6-6-05, Aramaki, Aoba-ku, Sendai 980-8579 Japan

## Abstract

Graphene nanoribbon (GNR), also known as 1-dimensional graphene, with a non-zero band gap has a huge potential for various electrical and optoelectrical applications because of its high transparency, flexibility, controllable band gap, and unique edge states. Recent advances in the synthesis of GNR enable us to show the possibility of GNRs as future high performance electrical devices. However, the applicability of GNRs to optoelectrical devices is unclear. Here we report that suspended GNR devices can show persistent photoconductivity (PPC) with long decay time (over 72 h) and adequate environmental stability. Repeated non-volatile memory operation is also demonstrated with an integrated PPC device using GNRs. This very stable PPC device can be applied to a wide variety of fields such as ultra-low-power non-volatile memory, nanoscale imaging, and biological sensors. Our results have opened the door to advance the study of GNRs in novel directions such as optoelectrical applications.

## Introduction

Graphene nanoribbon (GNR), a strip of graphene with nanometer width, can possess a non-zero band gap, thereby changing the electrical property of graphene from metallic to semiconducting behavior^1,2^. GNR is expected to be utilized in various electrical and optoelectrical applications because of its high optical transparency, mechanical flexibility, controllable band gap, and unique spin-polarized edge states^3–6^. Progress has been made in the synthesis of GNR such as bottom-up chemical synthesis of edge-controlled GNR^7–9^ and epitaxial synthesis of armchair GNR on a Ge substrate^10^. Although these advances enable us to demonstrate the possibilities of GNR in future high performance electrical devices, it is still a challenge to clarify the suitability of GNRs for optoelectrical applications because of the difficulty of GNR manipulation. Recently we have realized integrated synthesis of suspended GNRs with nanobar catalyst at the wafer scale^11,12^, which can offer a novel platform for GNR study to measure various optoelectrical properties of GNRs.

The current levels associated with persistent photoconductivity (PPC) can be modulated by photo-irradiation, and the modulated current can be maintained even after the photo-irradiation has ceased; this is a well known phenomena for bulk 3-dimensional (3D) materials^13–16^. PPC can be expected to be utilized for non-volatile memories^13,14^, imaging sensors^15^, and various chemical sensors^16^. Decreasing the size of the PPC device is important to increase the capacity of non-volatile memory, improve imaging resolution down to nanoscale, and develop other novel applications. Recently, it has been reported that PPC can be made to appear even in 2D materials by modifying the contact resistance between graphene and Au electrodes^17^ or by employing a heterojunction of graphene and molybdenum disulfide (MoS_2_)^18^. The modification of Au electrodes can show stable PPC in air but the decay time is very short (~5 s)^17^. Although the graphene/MoS_2_ heterojunctions can show a longer decay time, their environmental stability is relatively low because of the high chemical reactivity of MoS_2_ with various molecules such as oxygen, hydrogen, and water^18^. Fabrication of low-dimensional (low-D) PPC devices with high environmental stability is a crucial subject from the industrial point of view.

Here we report the successful fabrication of a highly stable PPC device with a suspended GNR array, which is grown by nanobar-catalyzed plasma CVD^11,12^. The functionalized GNR device shows pronounced PPC with a long decay time (over 72 h) and excellent room temperature stability in air. The PPC operation can be also realized even in water, indicating that GNR-based PPC devices can possess excellent environmental stability. By using these highly stable GNR-PPC devices, non-volatile memory operation was also demonstrated. The 1-bit device size can be decreased to ~0.25 μm^2^, and over 3960 GNR devices were integrated. The origin of the PPC was also systematically investigated. The very stable GNR-based PPC device has a huge potential for the realization of ultra-low-power non-volatile memory and various biological applications such as DNA sequencers in micro fluid devices^19^, *in vivo* imaging^20^, and microchip implants^21^. Our results demonstrate a novel direction of GNR study for optoelectrical applications.

**Figure 1:**
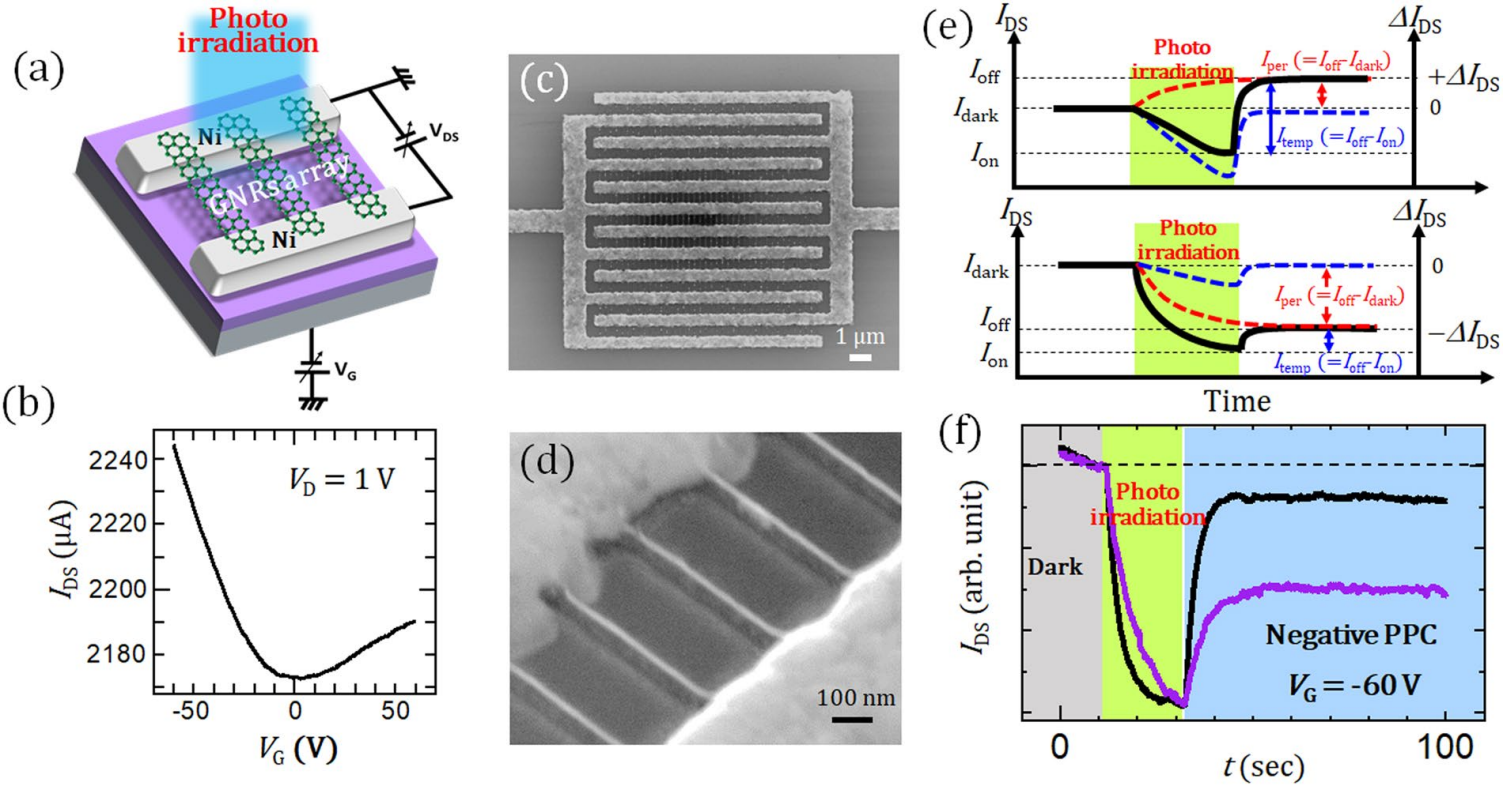
(**a**) Schematic illustration of GNRs FET. (**b**) Typical *I*_DS_ − *V*_G_ curve of a GNRs-array FET under *V*_DS_ = 1 V. SEM images of GNRs-array FET under (**c**) low and (**d**) high magnification. (**e**) Definition for *I*_dark_, *I*_on_, *I*_off_, *I*_per_, and *I*_temp_. Top and bottom show example of the case of positive and negative Δ*I*_DS_, respectively. (**f**) Typical photoresponse properties of fresh (black) and old (purple) GNR samples.

The GNR devices used in this studies were fabricated by plasma CVD with a Ni nanobar catalyst (Fig. 1a–d)^11,12^. The GNRs are about 10~30 nm in width, which was controlled by the initial Ni nanobar width. An image of a typical device is shown in Fig. 1a, where the field-effect transistor (FET) configuration of the suspended GNR device is formed with the source and drain electrodes made of Ni. The gate bias voltage (*V*_G_) was applied to the highly doped silicon substrate that had a 300 nm thick SiO_2_ layer as the gate insulator. Photo-irradiation was applied to this FET device by a solar simulator with light power (*P*) of 200 mW/cm^2^ unless otherwise specified. Typical spectrum of our light source (solar simulator) is shown in Fig. S1. A typical drain-source current (*I*_DS_) − *V*_G_ curve of this GNR-array device shows highly conductive ambipolar properties (Fig. 1b). Although the accurate layer number of GNR used in this study is not sure, it can be around 5 to 10 layers by judging from our previous studies.

## Results and Discussion

First, the photoresponse property of *I*_DS_ was measured for the GNR device immediately after the synthesis of the GNR; this will be referred to hereafter as a “fresh GNR device”. The deviation of *I*_DS_ (Δ*I*_DS_) between *I*_DS_ with light irradiation and without light irradiation (*I*_dark_) is defined as Δ*I*_DS_ = *I*_DS_ − *I*_dark_. The current levels of *I*_DS_ just before and after stopping photo irradiation in the steady state are defined as *I*_on_ and *I*_off_, respectively. In this study, we use two of important photo induced currents named persistent photoconductivity and temporal photoconductivity, which are defined as *I*_per_ = *I*_dark_ − *I*_off_ and *I*_temp_ = *I*_off_ − *I*_on_, respectively (Fig. 1e). The fresh device shows the usual photoresponse, where current suddenly decreases upon irradiating the device with light, and then the current level goes back to the original value after stopping the light irradiation (black curve in Fig. 1f). This means the *I*_per_ is close to 0. Next, a similar measurement was carried out for an “old” GNR device that had been stored in air for several months. The old device also shows a similar photoresponse which is temporal depression of *I*_DS_ during the light irradiation. Interestingly, the current level did not go back to the original value, and stabilized at a certain level even after stopping the light irradiation (Fig. 1e and purple curve in f). This is characteristic of PPC behavior^13–16^. Similar phenomena have been reported for other 2D materials such as suspended graphene with oxidized Au electrodes^17^ and heterojunctions of graphene and MoS_2_^18^.

**Figure 2:**
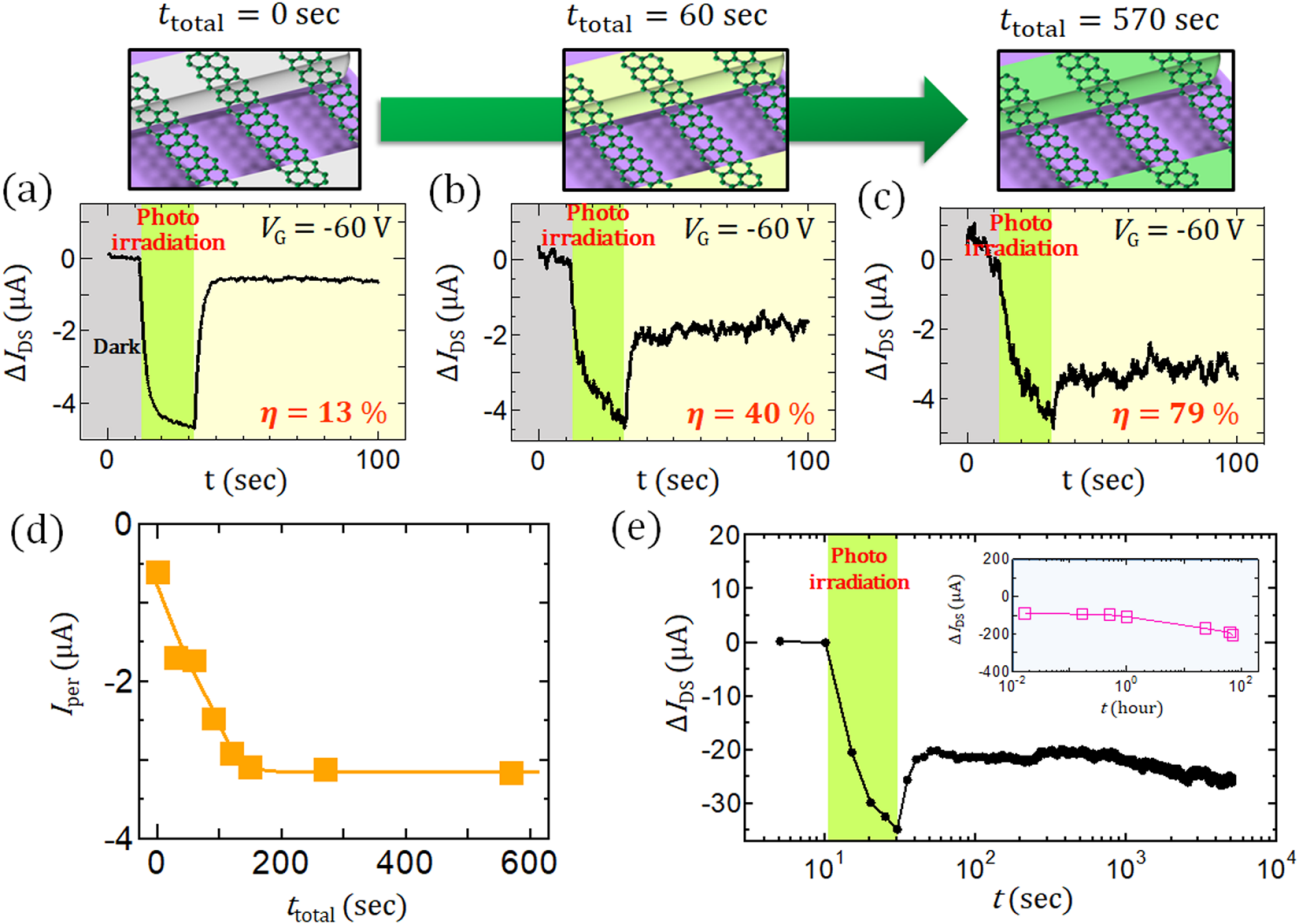
(**a**–**c**) Photoresponse of Δ*I*_DS_ with different total mild O_2_ plasma treatment times (*t*_total_) (*t*_total_ = (**a**) 0, (**b**) 60, (**c**) 570 sec). (**d**) Plot of *I*_per_ as a function of *t*_total_. (**e**) Continuously measured time profile of *I*_DS_ for an old GNR sample up to ~4000 sec. The inset shows a plot of Δ*I*_DS_ measured for a total of 3 days. All of the data in this figure were taken under *V*_DS_ = 1 V, *V*_G_ = −60 V.

To elucidate the difference between fresh and old GNR devices, we introduced an oxidation process to the fresh device. Oxidation was carried out with mild oxygen (O_2_) plasma treatment. It was found that *I*_per_ gradually increases with an increase in the plasma irradiation time and obvious PPC can be observed that is similar to that of the old GNR device (Fig. 2a–d). Similar results can be also observed after simple annealing in air at 350 °C (Fig. S2). This indicates that the PPC that appeared in the old GNR device should be due to the oxidation of Ni or related reactions; this is similar to the previously reported graphene and oxidized-Au results^17^. It is noteworthy that the *I*_per_ for the GNR with oxidized-Ni electrode can maintain its current level more than 3 days (72 h), which is about 25,000 times longer than that of graphene with oxidized-Au electrodes (Fig. 2e).

**Figure 3:**
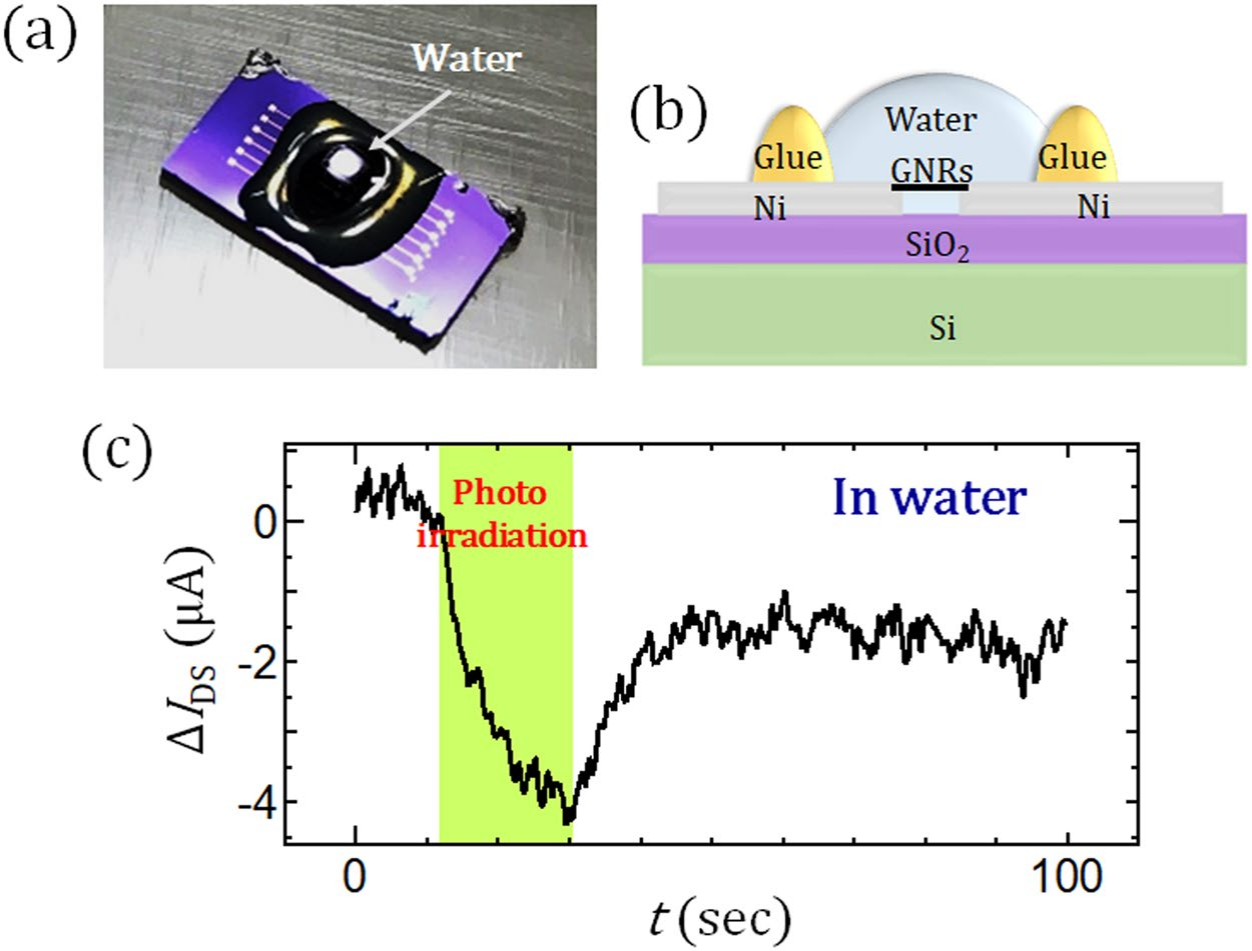
(**a**,**b**) (**a**) Optical microscope image and (**b**) Schematic illustration of PPC measurement in water. (**c**) Typical time profile of *I*_DS_ for a GNR measured in water.

Using this very stable PPC device, similar measurements were also carried out in water. The GNR device was covered with a droplet of water, and *I*_DS_ was measured with and without photo-irradiation (Fig. 3a and b). Surprisingly, obvious *I*_per_ can be also observed even in water with excellent stability (Fig. 3c). This indicates that the GNR-based PPC device has very high environmental stability and can be used for various biological applications such as DNA sequencers in micro fluid devices^19^, *in vivo* imaging^20^, and microchip implants^21^.

**Figure 4:**
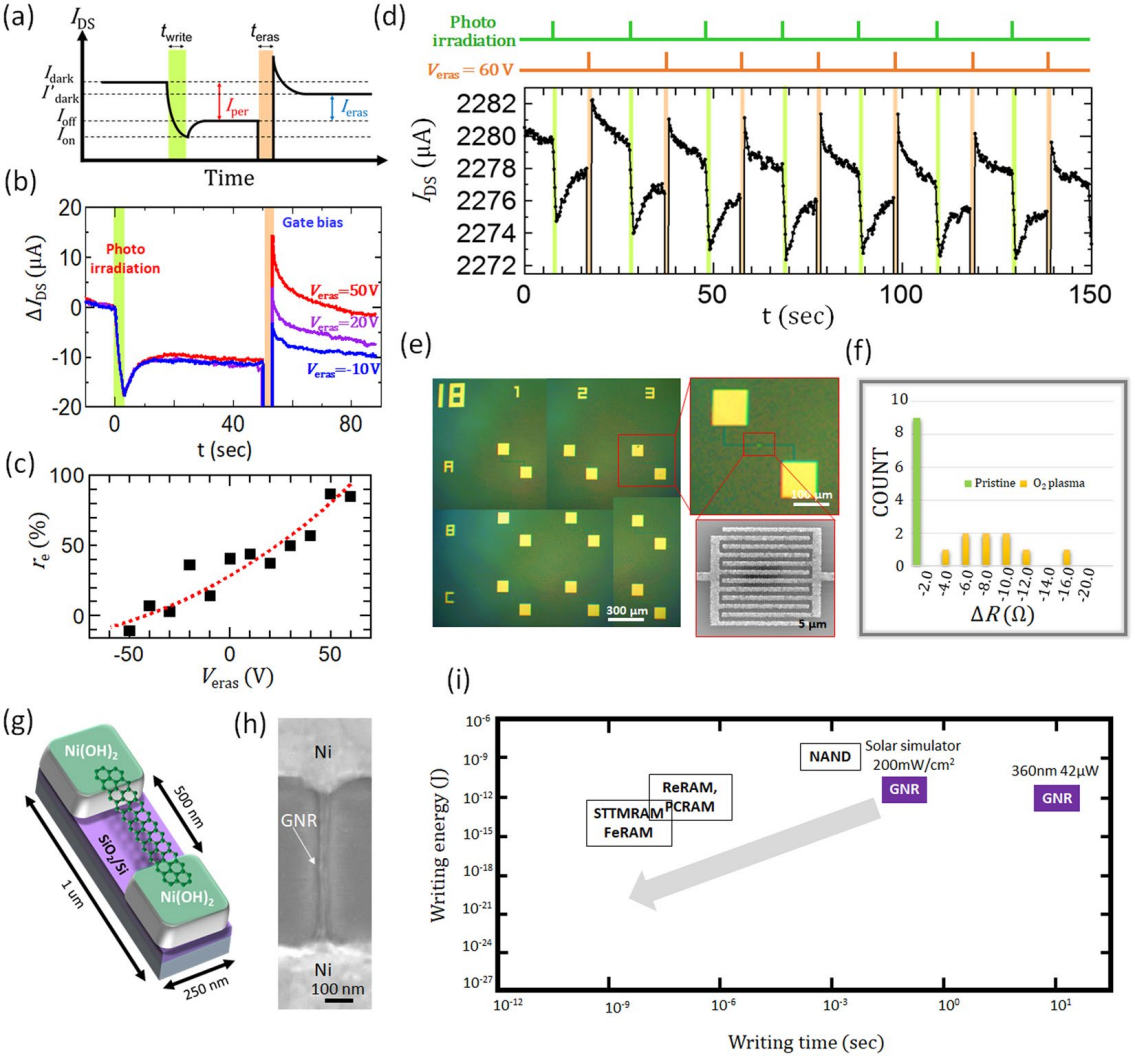
(**a**) Typical time sequence of wiring and erasing operation. Definition of dark current after erasing (*I*′_dark_) and erasing current (*I*_eras_) are shown as arrows. (**b**) Writing and erasing operation process with different values of the erasing bias voltage (*V*_eras_). (**c**) The dependence of the recovery rate (*R*_e_) on *V*_eras_. (**d**) Repeated operation (8 cycles) of optical memory, timing of photo illumination (green) and application of *V*_eras_ (orange) is shown in the upper graph. (**e**) Low and high magnification optical microscope images and SEM image of the integrated GNR memory (9 cells). Each cell includes 440 GNRs. (**f**) Plot of the resistance change before and after photo-irradiation for pristine (green) and mild O_2_-plasma-treated 9-cell GNR devices (yellow). (**g**,**h**) (**g**) Schematic illustration and (**h**) typical SEM image of a single-GNR device. (**i**) Comparison of device specifications between our GNR device and other memory devices. The arrow denotes the future possibilities of our GNR device.

Since very stable PPC can be observed with our GNR device, we attempted to demonstrate memory operation. For the practical application of any memory device, it is necessary to realize three main operations: writing, reading, and erasing. The creation of stable *I*_per_ as demonstrated above shows that the writing operation is possible by irradiating the device with light. The writing information can be read by the deviation of current through the GNRs. Since the origin of the *I*_per_ can be attributed to the trapped charge created by light irradiation, we attempted to apply a pulsed gate voltage to the GNR device to release the trapped charge (the detailed mechanism of *I*_per_ will be discussed later). Here, the erasing rate is defined as *r*_e_ = 100 × *I*_eras_/*I*_per_, where *I*_per_ = *I*_off_ − *I*_dark_, and *I*_eras_ = *I*′_dark_ − *I*_off_ (Fig. 4a). The pulse width for erasing (*t*_eras_) was set to 3 sec. When *V*_G_ is switched from *V*_G_ = −60 V to positive *V*_G_, Δ*I*_DS_ temporally increases then decreases and stabilizes after the *V*_G_ is back to original value (−60 V). The Δ*I*_DS_ of the stable state clearly decreases with an increase in *V*_G_ and reaches zero when *V*_G_ = 50 V, where *r*_e_ reaches around 90% (Fig. 4b and c). The detailed erasing mechanism will also be discussed later. This indicates that the erasing operation can be accomplished by applying a relatively high pulsed gate bias voltage. Repeated operations of writing, reading, and erasing were also demonstrated (Fig. 4d). These periodic changes of *I*_DS_ can be produced repeatedly, showing that our GNR device can operate as a non-volatile memory with high stability under normal atmosphere and room temperature. For a practical optical memory device, integration of the memory device is very important to improve the storage ability. With this in mind, an integrated GNR memory has been fabricated as shown in Fig. 4e–h, where 9 memory cells were fabricated, and each memory cell contains 440 GNR arrays. We measured the differential resistance Δ*R* = *R*_after_ − *R*_before_; here *R*_before_ and *R*_after_ are the resistances of the GNR arrays before and after photo-irradiation for all 9 cells. A pronounced change of Δ*R* was observed in all the cells after a mild O_2_ plasma treatment (Fig. 4f). This shows that all of the memory cells can function as a non-volatile memory. In one memory cell, 440 GNRs are integrated with high integration density (0.25 µm^2^) (Fig. 4e). It is also confirmed that the PPC can appear not only for the GNR array, but also for a single GNR device (Figs 4g,h and S3). This indicates that each GNR device can work as a single-bit memory. The memory size for 1 bit of storage can be given by a simple calculation, which shows that the 1-bit memory size can be decreased down to 0.25 µm^2^ with our single GNR device (Fig. 4g and h). This scale advantage implies that 0.4 Gbits of memory can be integrated within 1 cm^2^. The GNR memory also has the advantage of low energy operation. Figure 4i summarizes the writing energy vs. writing time for various memories. The NAND memory that is widely used in practical devices needs a writing energy of 10^−9^ J and its speed is about 10^−3^ sec^22^. Recent advances in memory technology can lower the writing energy and increase the speed using novel operational principles such as PCRAM, ReRAM, STT-MRAM, FeRAM, and SRAM^22^. When we plot the specifications of our GNR memory in Fig. 4i, the potential abilities of GNR memory can be elucidated. Because of the limitations of the measurement system, the writing speed of our GNR memory was set as 0.1 sec (Fig. S4). If we had used a writing speed of 10 ns, which is similar to that of FeRAM, the writing energy of GNR memory could be decreased down to 5 × 10^−21^ J, which is about 10^−7^ times lower than that of FeRAM^22^. It has been reported that the photoresponse time can be decreased to 5.5 ps with graphene/TMD devices^23^, indicating that the assumption of 10 ns operating time for the GNR memory should be reasonable.

**Figure 5:**
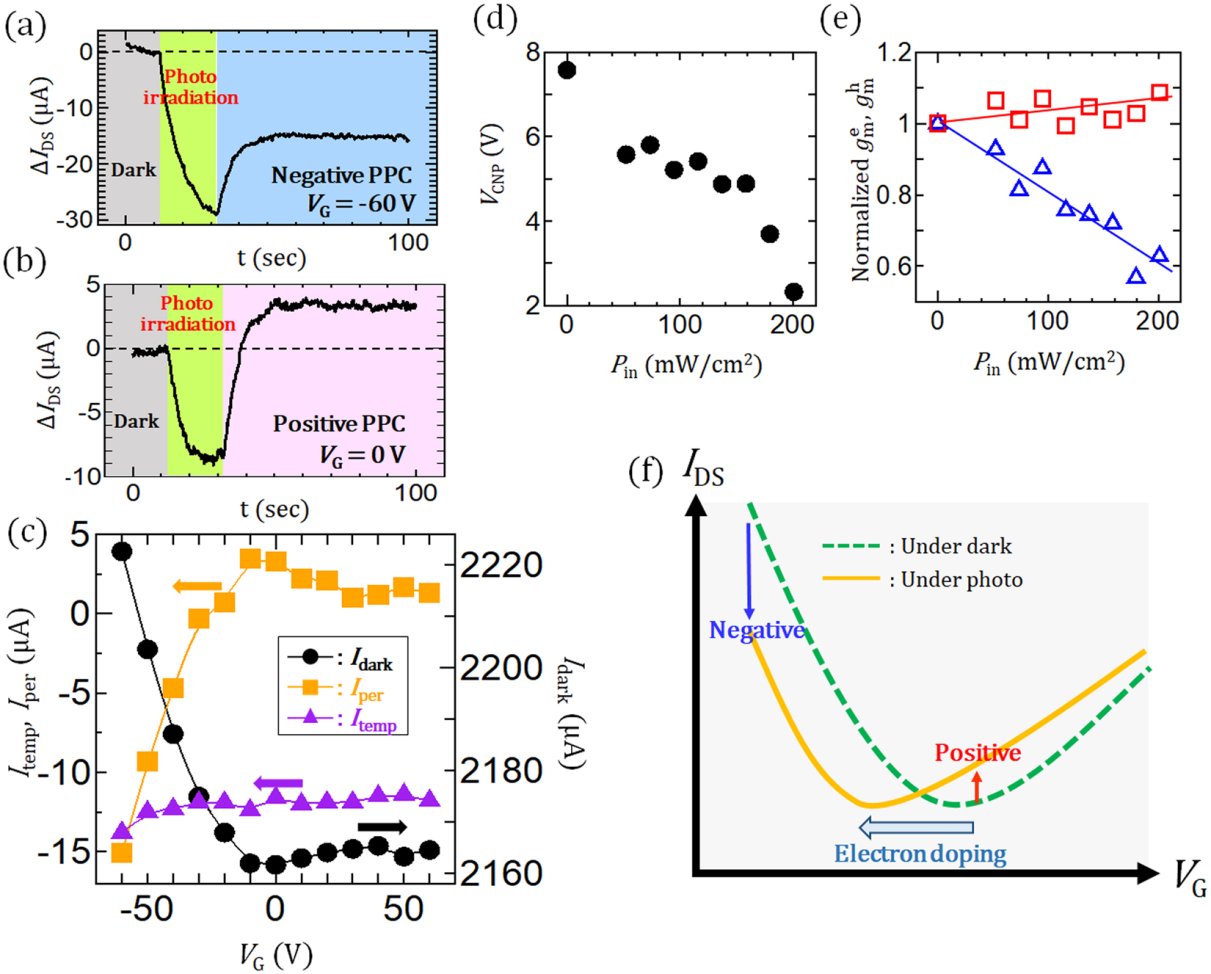
(**a**,**b**) Time resolved photoresponse of Δ*I*_DS_ under (**a**) *V*_G_ = −60 V, (**b**) *V*_G_ = 0 V under *V*_DS_ = 1 V. (**c**) *V*_G_ dependency of temporary photocurrent (*I*_temp_), persistent photocurrent (*I*_per_), and *I*_dark_ under *V*_DS_ = 1 V. (**d**,**e**) Light power (*P*) dependence of (**d**) charge neutral point (*V*_CNP_) and (**e**) transconductance of electrons (g_m_^e^) (blue triangle) and holes (g_m_^h^) (red square) normalized by the dark value under *V*_DS_ = 1 V. (**f**) Schematic illustration of *V*_G_ − *I*_DS_ curve under dark (green) and (yellow) photo-irradiation.

To further improve device performance, it is necessary to fully understand the operating mechanism. For this reason, detailed PPC measurements for the array of GNRs were carried out with under different values of *V*_G_ (*V*_G_ = −60 V or 0 V) (Fig. 5a and b). As already discussed above, *I*_temp_ can produce a negative value of Δ*I*_DS_ during photo-irradiation. This is observed with high reproducibility even under high vacuum conditions, indicating that the adsorption or desorption of impurities on the surface of the GNR caused by photo-irradiation can be ignored in our GNR devices. The origin of this *I*_temp_ can be explained by considering the conductance change during photo-irradiation. It has been confirmed that the conductance of our GNR device decreases with increasing temperature, denoting a negative bolometric coefficient around 300 K (Fig. S5)^24,25^. Thus, the temperature increase of GNR caused by photo-irradiation should be the critical origin of *I*_temp_. For the *I*_per_ component, an obvious change of the polarity can be observed for different values of *V*_G_. The negative and positive *I*_per_ can be observed with *V*_G_ = −60 V (Fig. 5a) and *V*_G_ = 0 V (Fig. 5b), respectively. The dependencies of *I*_temp_ and *I*_per_ on *V*_G_ are plotted in Fig. 5c. We can see that *I*_per_ strongly depends on *V*_G_ and can be tuned between negative and positive levels by changing *V*_G_ from −60 V to +60 V, whereas *I*_temp_ hardly depends on *V*_G_. The weak *V*_G_ dependence of *I*_temp_ is consistent with the explanation based on the bolometric effect. To elucidate the origin of the *V*_G_ dependence of *I*_per_, the *I*_DS_ − *V*_G_ curve was measured with and without light irradiation. A clear shift of the charge neutral point (*V*_CNP_) in the negative *V*_g_ direction can be observed during photo-irradiation. The value of the shift increases with the light power (*P*_in_) (Fig. 5d). The transconductance of electrons (g_m_^e^) decreases (blue triangle in Fig. 5e) while that of holes (g_m_^p^) hardly changes with increasing *P*_in_ (red square in Fig. 5e). These results imply that GNRs are n-doped by photo-irradiation, and the doped carriers may act as scattering centers for electrons, resulting in the decrease of g_m_^e^. Therefore, the negative and positive persistent current can be observed with *V*_G_ = −60 V and 0 V, respectively (Fig. 5f). Carrier doping effects caused by photo-irradiation have been reported in graphene devices, and the mechanism is the photo-gating effect that originates in charging of the SiO_2_ surface during photo-irradiation^24,25^; consequently, it can be assumed that hot carriers excited by light irradiation can be deeply trapped at specific sites, causing a very stable *I*_per_ in our GNR devices. Note that the minimum power of solar simulator for the appearance of PPC was ~25 mW/cm^2^, which is several orders higher than that of other previous results^15,17^. This is because of the effect of wavelength dependence. Only ultraviolet (UV) light region is effectively used for PPC generation (discussed later).

As discussed above, if *I*_per_ originates from the trapped charge, stable trap sites should be formed in our GNR device by mild O_2_ plasma treatment. To identify the effects of mild O_2_ plasma treatment on the formation of trapping site, following systematic experiments were carried out.

### Effects of mild O_2_ plasma treatment on the GNR structure

First, we try to identify the effect of mild O_2_ plasma treatment to GNR structures. Raman scattering spectroscopy was used to identify the introduction of defects to GNR. Note that because it is very difficult to consider the edge effect of GNR, we used mechanically-exfoliated graphene instead of GNR for this Raman experiment. For the pristine graphene transferred on the Ni (50 nm)/SiO_2_/Si substrate, repeatable mild O_2_ plasma treatments were carried out. It is found that D-band peak relating with the disorder of graphitic structure can be observed even after 1 min plasma treatment (Fig. S6). The ratio of D-band to G-band (I_D_/I_G_) gradually increased with plasma treatment time. The introduction of defects was more significant for the thinner graphene. This indicates that the defects should be introduced to GNR by our mild O_2_ plasma treatment. It should be noted that even after long time plasma treatment (5 min), over all film structure did not change (Fig. S6), denoting not the etching reaction but introduction of local defects should be dominant in our mild O_2_ plasma treatment. This is because of the low ion energy in mild plasma reaction, which had been already developed by our previous study^26^.

### Effects of mild O_2_ plasma treatment on the Ni electrode

To identify the effects of plasma treatment on the Ni electrode, detailed analysis was carried out. The candidates for the functionalized structures of Ni are NiO, Ni_2_O_3_, or Ni(OH)_2_, where the band gap has been reported to be about 4.3 eV^27^, 3.38 eV^28^, and 3.6–3.9 eV^29^ respectively. The wavelength (λ) dependence of the photoresponsivity *R*_p_ (=Δ*I*_DS_/*P*_in_*S*) was measured for the old GNR device, whereΔ*I*_*DS*_, *P*_in_, and *S* denote current change before and after light irradiation, incident light power, and the area of the GNR channel, respectively. The λ-selective irradiation was carried out by splitting the light source from a Xe lamp with a conventional spectrometer. A clear λ dependence of *R*_*p*_ was observed, and only irradiation at UV wavelength would be capable of producing such a high value of *R*_p_ (Fig. S7). With the light of 360 nm wavelength, PPC can be observed even with the low power of 41 μW/cm^2^. The optical adsorption spectra were measured for Ni thin films deposited on a quartz substrate without graphene layer before and after the mild O_2_ plasma treatment. Mild O_2_ plasma treatment is carried out for 10 min with homemade plasma CVD system (See method). The adsorption spectra show obvious adsorption in UV region, but this appears only for mild O_2_-plasma-treated Ni films (Fig. S8). This is consistent with the λ dependence of *R*_p_, suggesting that the dominant trapping sites are not oxidized GNR (band gap: 0.02~2 eV^30^), but rather, oxidized or hydroxidized Ni. To further identify the detailed structure of functionalized Ni serving as the trapping site, X-ray photoelectron spectroscopy (XPS) measurements were carried out. After mild O_2_ plasma treatment (5 min), the peak of pure Ni vanished and only the chemical-shift peak was observed at 855.69 eV which is obviously different from that of NiO (854.0 ± 0.5 eV) and very close to that of Ni_2_O_3_ (856.6 ± 0.8) and Ni(OH)_2_ (855.7 ± 0.4) (Fig. S9a)^31^. The shapes of the peaks over a wide range of spectrum were also taken into consideration to judge the origin of the peak at 855.69 eV. The observed spectrum after mild O_2_ plasma treatment is well matched with that of Ni(OH)_2_ (Fig. S9b)^32^. Atomic force microscopy (AFM) measurements were also performed to obtain structural information about the functionalized Ni after mild O_2_ plasma treatment (5 min). Before mild O_2_ plasma treatment, the surface of the Ni is relatively smooth, and its average of roughness height (*R*_a_) is 0.05 nm (Fig. S10a and b). On the other hand, after mild O_2_ plasma treatment, surface becomes rough (*R*_a_ = 0.59 nm) and a honeycomb-like nanostructure is formed (Fig. S10c and d) with ~1 nm height and ~10 nm width (Fig. S10d). A similar layered structure has been reported for Ni(OH)_2_ (β-Ni(OH)_2_)^33^. Judging from this systematic analysis, it can be concluded that β-Ni(OH)_2_ nanostructures are probably formed on the surface of Ni by mild O_2_ plasma treatment.

### Interlayer structure between GNR and Ni after mild O_2_ plasma treatment

Because it is revealed that mild O_2_ plasma treatment modify GNR and Ni surface to disordered GNR and β-Ni(OH)_2_, respectively, we have to think about the effects of those modified structures on the appearance of PPC. If the disorder site of GNR itself is the dominant trapping site of carrier, PPC should be observed independent from electrode materials. Then, we fabricated similar suspended-graphene device with various electrode materials such as Ni, Au, and Cu by transferring mechanically-exfoliated few-layer graphene to electrodes. Interestingly, PPC can be observed only for Ni electrodes after mild O_2_ plasma treatment (Fig. S11). This indicates that kinds of electrode materials possess significant contribution to determining the appearance of PPC, i.e. forming defects to channel region of GNR is not enough to cause PPC.

Then, we attempt to confirm the effects of β-Ni(OH)_2_. Similar suspended-graphene device was fabricated (Fig. S12). Before the transfer of graphene, Ni electrode was pre-oxidized by mild O_2_ plasma treatment. Interestingly, current can not be through in this pre-oxidized Ni/GNR device, which should because of the poor contact between pre-oxidized Ni and GNR. By considering this point, it can be conjectured that surface of Ni underneath of GNR is not completely oxidized but partial oxidation can happen for the device where PPC can be observed (Fig. S13). Such local structures of disordered GNR/β-Ni(OH)_2_ can dominantly work for the appearance of PPC. Very stable trapping site may be formed at such modified structures. As a control experiment, we also carried out similar experiments with not suspended but supported graphene device with Ni electrode. Noticeably, however, neather PPC nor temporal response can not be observed (Fig. S14). Because of the bottom contact structure of graphene and Ni electrode, it should be difficult to form local structures (disordered graphene and Ni(OH)_2_) at the contact region between graphene and Ni for supported graphene device, which can be a possible explanation for the lack of PPC features in the supported graphene device. This indicates that the top contact of Ni and GNR should be important to cause PPC and suspended GNR devices can effectively provide such ideal contact structures for PPC.

### Possible model for appearance of PPC in GNR device

**Figure 6:**
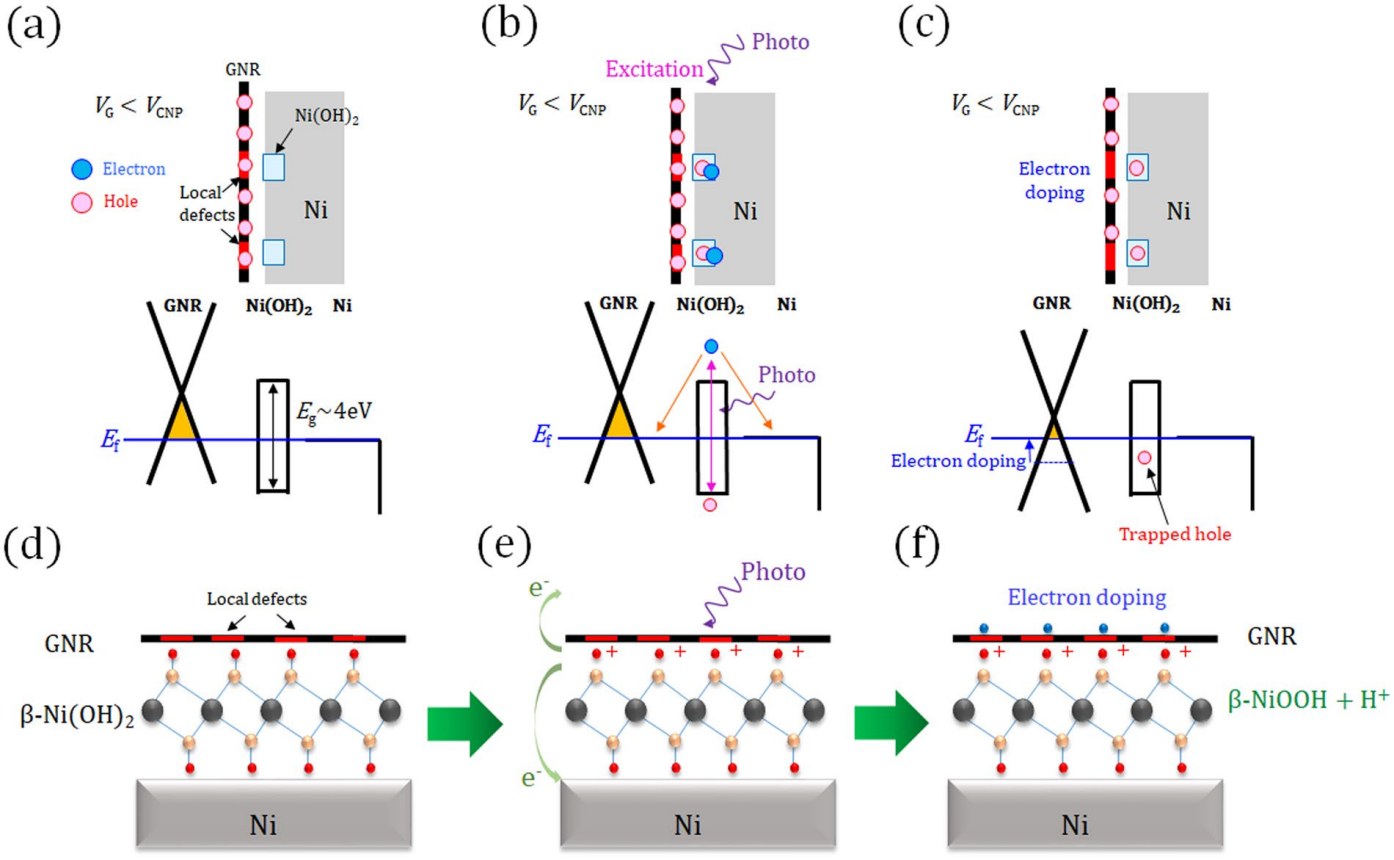
(**a**–**c**) Schematic illustration and (**d**–**f**) charge-trapping mechanism of GNRs-Ni(OH)_2_ system for (**a**,**d**) before, (**b**,**e**) during photo-irradiation and (**c**,**f**) after the application of *V*_eras_.

Based on these results, we can propose the following mechanisms of PPC in our GNR device (Fig. 6). As discussed above, local defects are introduced to GNR by mild O_2_ plasma treatment. The local defect can enhance the formation of nanoscale layered β-Ni(OH)_2_ structures around the defects site. Then, locally modified structure of disordered GNR/β-Ni(OH)_2_ should be formed at the interface between the GNRs and the Ni electrodes (Fig. 6a). When photo-irradiation is directed at this system under an applied *V*_G_ < *V*_CNP_ (Fig. 6b), electrons can be optically excited in disordered GNR/β-Ni(OH)_2_ and relaxed by going through Ni or GNR resulting in the creation of a localized hole. If this localized hole is trapped at a stable site at the interface of disordered GNR and β-Ni(OH)_2_, the hole can generate electrons in the GNR because of Coulomb interactions, resulting in the fact that electron doping in GNRs can be induced by photo-irradiation (Fig. 6c). The modification of the electronic structure of graphene is known to be not limited only around the metal electrodes but extends 200~300 nm into the graphene channel^34^. By applying a positive *V*_G_ (*V*_G_ > *V*_CNP_), recombination of electrons and holes can be initiated, causing the erasing operation as shown in Fig. 4b and c. Protonation can be considered as one of the possible candidates for the origin of hole generation. It is known that β-Ni(OH)_2_ is easily decomposed to β-NiOOH + H^+^ by extracting an electron. Hence, if an electron is extracted from Ni(OH)_2_ by photo-excitation, the surface of the β-Ni(OH)_2_ may change to β-NiOOH + H^+^. This kind of surface protonation has been reported in other materials^35^.

We mainly discussed the stable and long-decay time of PPC as a performance of our GNR device. As a one of the important performance of PPC, the conductivity-switching effect is also reported^35^. Since the GNRs used in our study is relatively thicker and wider geometry, only weak conductivity switching was observed. By using further thinner and narrower GNRs including high on/off current ratio, large conductivity-switching effects can be also expected with GNR device, which can be significant difference compared with graphene-based PPC device.

In summary, we have fabricated very stable PPC devices with suspended GNRs. The stable PPC can be obtained not only in air but also in a solution phase. Optically-driven non-volatile memory operation has been also demonstrated with these stable PPC devices with suspended GNRs. The GNR memory device can be densely integrated, and minimum memory size for 1 bit of storage can be decreased to 0.25 μm^2^. The energy for writing operation can be as low as 0.1 pJ/bit, indicating that the GNR-based non-volatile photo memory has a huge potential for practical use in future low power electronics. The detailed operation mechanism was also investigated, showing that the heterojunction between locally-disordered GNR and nanoscale β-Ni(OH)_2_ structures can behave as a stable trapping site for optically generated holes, causing the excellent stability of the PPC.

## Methods

### Plasma CVD

A homemade plasma CVD system was used for the rapid-heating plasma CVD (RH-PCVD). Before the plasma CVD growth, an electric furnace was heated to the desired temperature (typically 800–900 °C) under flowing hydrogen (50 Pa). A substrate was immediately transferred to the center area, and rapid heating was performed. CH_4_ and H_2_ gases at a 9:1 ratio (250 Pa) were inletted immediately after a pre-set temperature was reached. Next, radio frequency power (100 W, 13.56 MHz) was supplied to the coils outside of the quartz tube.

The plasma was typically maintained for 5–30 s. Following the plasma CVD, the substrate was moved from the center to the outside of the electrical furnace so that its temperature would rapidly decrease.

### Mild O_2_ plasma treatment

A homemade plasma treatment system was used. An mild O_2_ plasma can be generated by supplying radio frequency power (26 W, 13.56 MHz) to the coils outside of the quartz tube under flowing O_2_ gas (150 sccm). A grid mesh was set 15 cm ahead of the center of the coil. The sample was 40 cm away from the center of the coil.

### Characterizations

The structure of the GNR array sample was characterized by scanning electron microscopy (SEM; Elionix, ELS-7500EXTK and Hitachi, SU1510, Japan). The electrical measurements of the GNR devices were performed using a vacuum probe station with a semiconductor parameter analyzer (HP 4155C). The elemental analysis of the Ni film was characterized by X-ray Photoelectron Spectroscopy (XPS; Ulvac-phi, ESCA1600, Japan). The structure of the Ni surface was analyzed by atomic force microscopy (AFM; JEOL, JSPM-5400, Japan).

## Electronic supplementary material

Supplementary dataset
