# Supplementary material for: Highly Stable Persistent Photoconductivity with Suspended Graphene Nanoribbons
Source: Sci Rep. 2018 Aug 7;8:11819. doi: 10.1038/s41598-018-30278-z (PMC6081463; doi:10.1038/s41598-018-30278-z)
Supplement: Supplementary file 1 — Supplementary dataset [file 41598_2018_30278_MOESM1_ESM.docx]

**Supplemental data set**

Highly Stable Persistent Photoconductivity with Suspended Graphene Nanoribbons

Hiroo Suzuki, Noritada Ogura, Toshiro Kaneko and Toshiaki Kato*


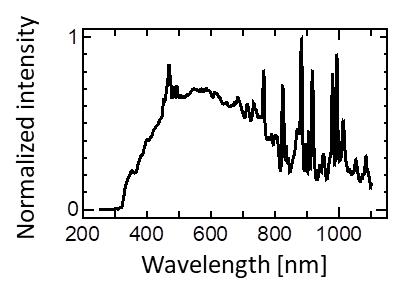


***Figure S1.*** Typical spectrum of light source (solar simulator) used in this study for the PPC measurement.


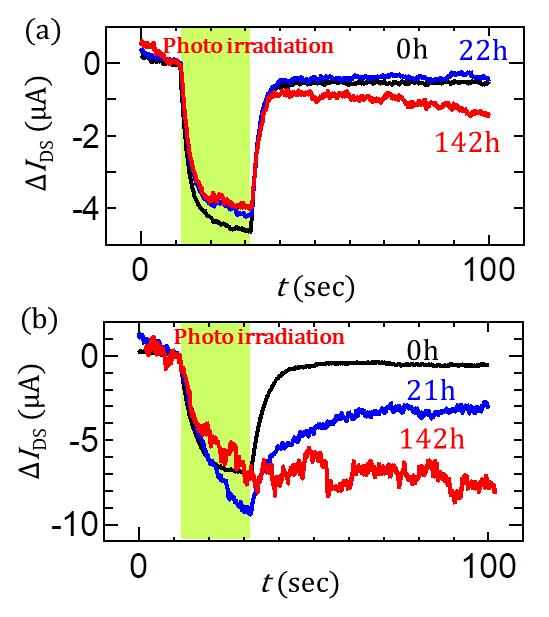


***Figure S2.*** Typical time profile of Δ*I*_DS_ of a suspended GNR device annealed in air at different temperatures [(a)100 ºC and (b) 350 ºC].


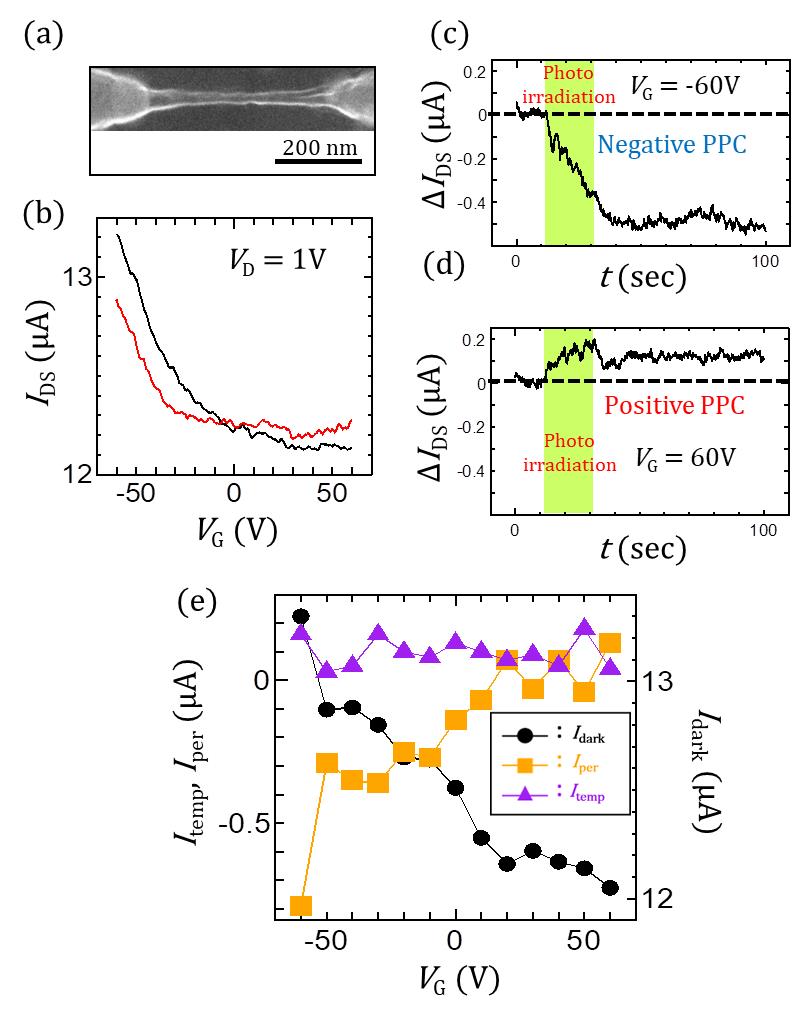


***Figure S3.*** PPC features obtained from a single GNR device. (a) Typical SEM image of a single GNR used for this PPC measurement. (b) *I*_DS_-*V*_G_ curves of a single GNR without (black) and with (red) irradiation. (c,d) Time profile of Δ*I*_DS_ under different *V*_G_ conditions [(c) *V*_G_ = −60 V and (d) *V*_G_ = +60 V]. (e) Dependence of *I*_dark_ (black), *I*_per_ (yellow), and *I*_temp_ (purple) on *V*_G_.


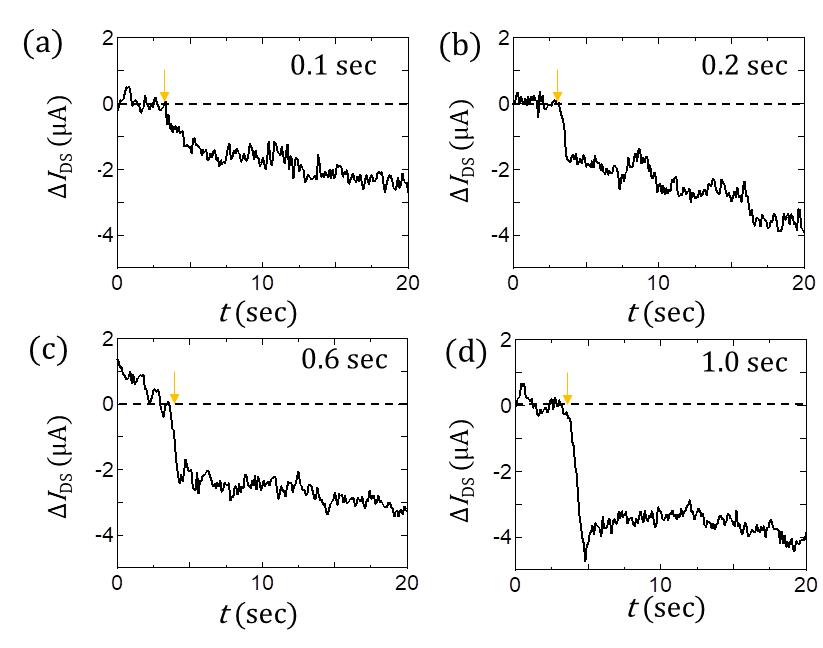


***Figure S4.*** Typical time profiles of Δ*I*_DS_ under the different photo irradiation time ((a) 0.1sec, (b) 0.2 sec, (c) 0.6 sec, (d) 1.0 sec). The arrows show the timing (start and stop) of photo irradiation.


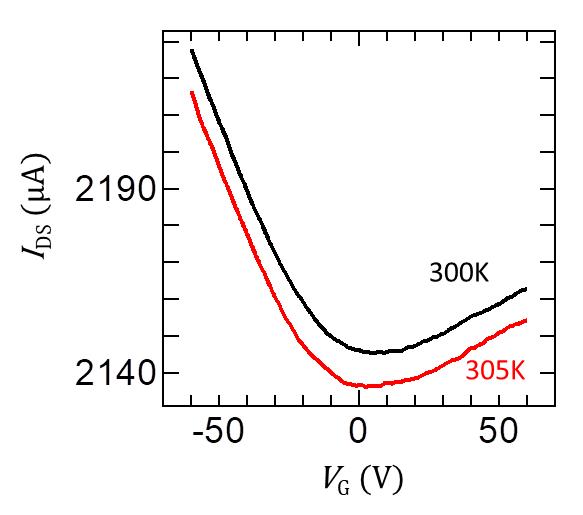


***Figure S5.*** Typical *I*_DS_-*V*_G_ curves of suspended GNRs measured under different temperatures [300 K (black) and 305 K (red)].


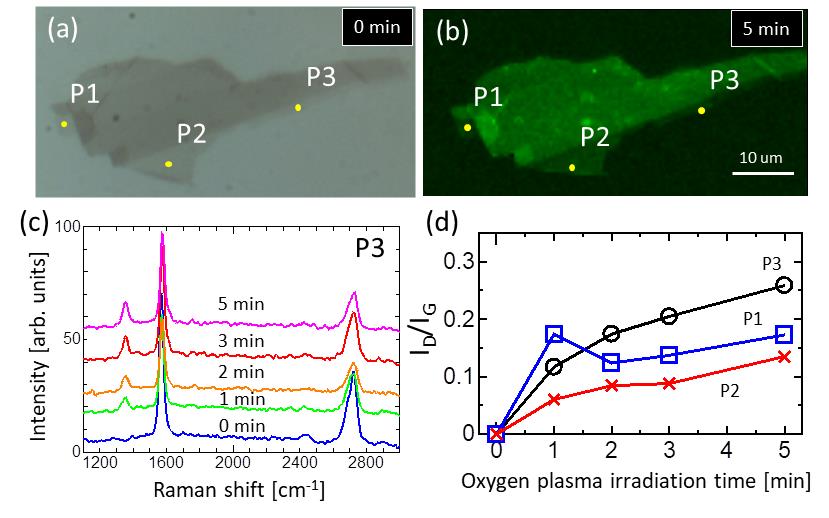


***Figure S6.*** Effects of oxygen plasma treatment on graphene structure. (a) Optical microscope image of mechanically exfoliated graphene before plasma treatment. (b) G-band Raman intensity mapping of graphene after 5 min oxygen plasma treatment. (c) Dependence of plasma irradiation time on the raw Raman scattering spectra of graphene measured at point 3 (P3). (d) Time dependence of I_D_/I_G_ for different point (P1, P2, P3). The thickness of graphene layer varies for each position (P1, P2, P3). The order of absolute G-peak intensity is P2 > P1 > P3, denoting layer number of graphene should be P2 > P1 > P3.


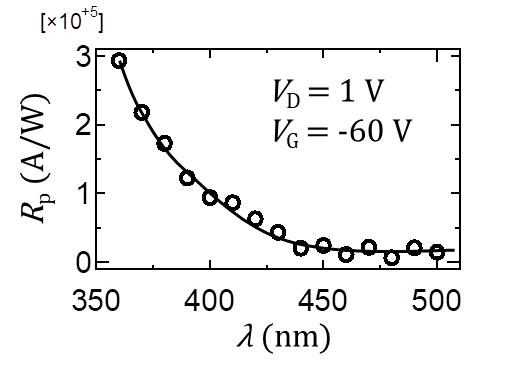


***Figure S7.*** λ dependence of *R* in a suspended GNR.


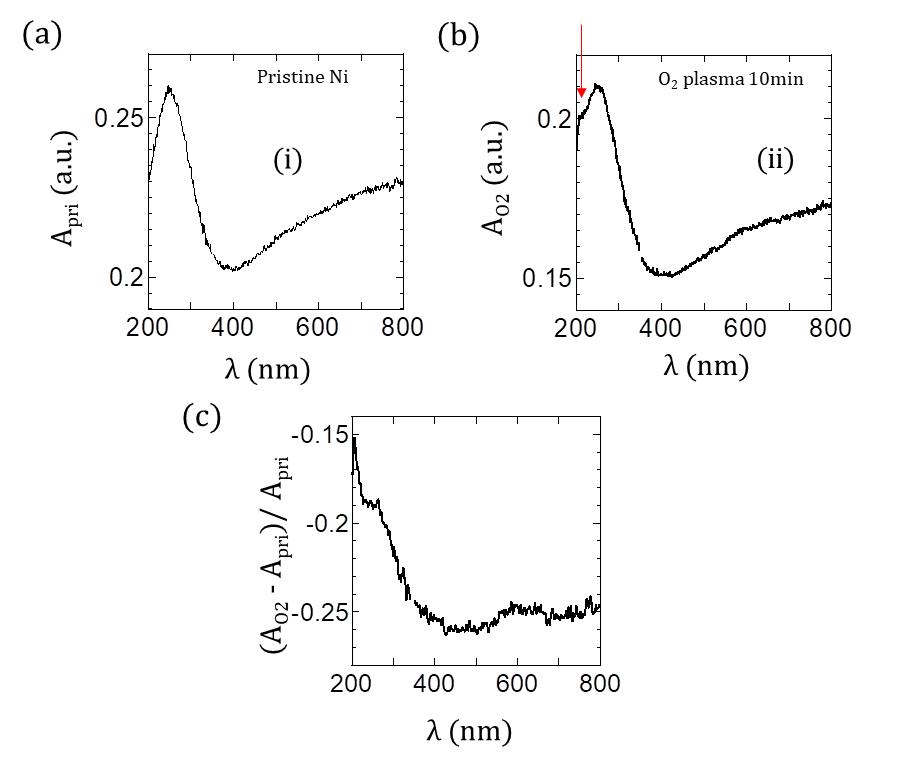


***Figure S8.*** Typical absorption spectra of (a) pristine (A_pri_) and (b) O_2_-plasma-treated (A_O2_) thin Ni films. (c) Normalized absorption spectrum [(A_O2_ − A_pri_ )/A_pri_].


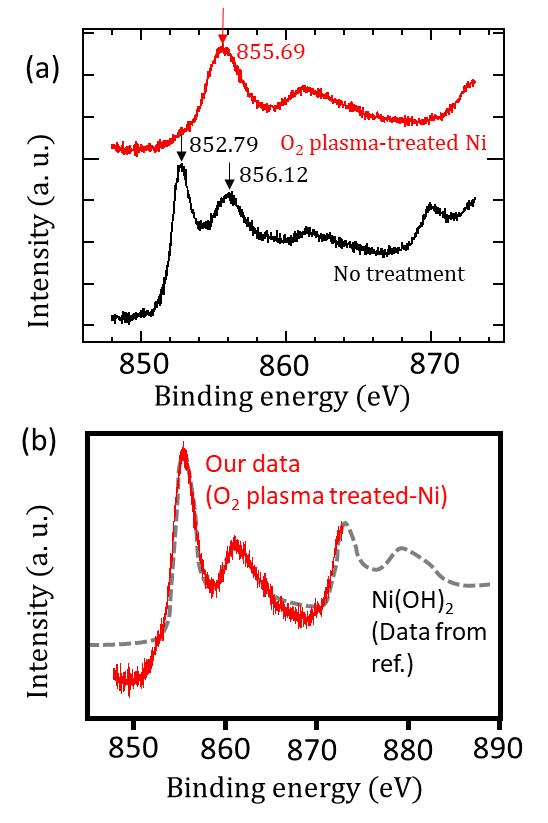


***Figure S9.*** (a) XPS spectra of pristine (black) and O_2_-plasma-treated (red) thin Ni films. (b) Comparison of wide range XPS spectra between O_2_-plasma-treated thin Ni films (red) and Ni(OH)_2_ (gray) traced from ref [Su, Y.-Z. *et al.*, *J. Mater. Chem. A* **2014**, *2*, 13845–13853].


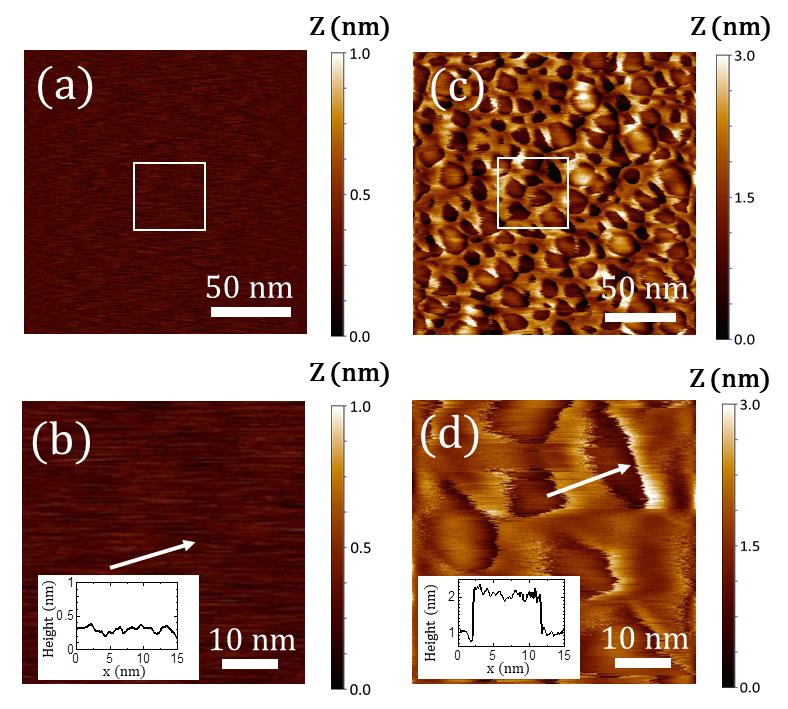


***Figure S10.*** (a,c) Low and (b,d) high magnification AFM images of (a,b) pristine and (c,d) O_2_-plasma-treated thin Ni films. The insets in b and d show the height profiles of the Ni surface along the arrows of b and d, respectively.


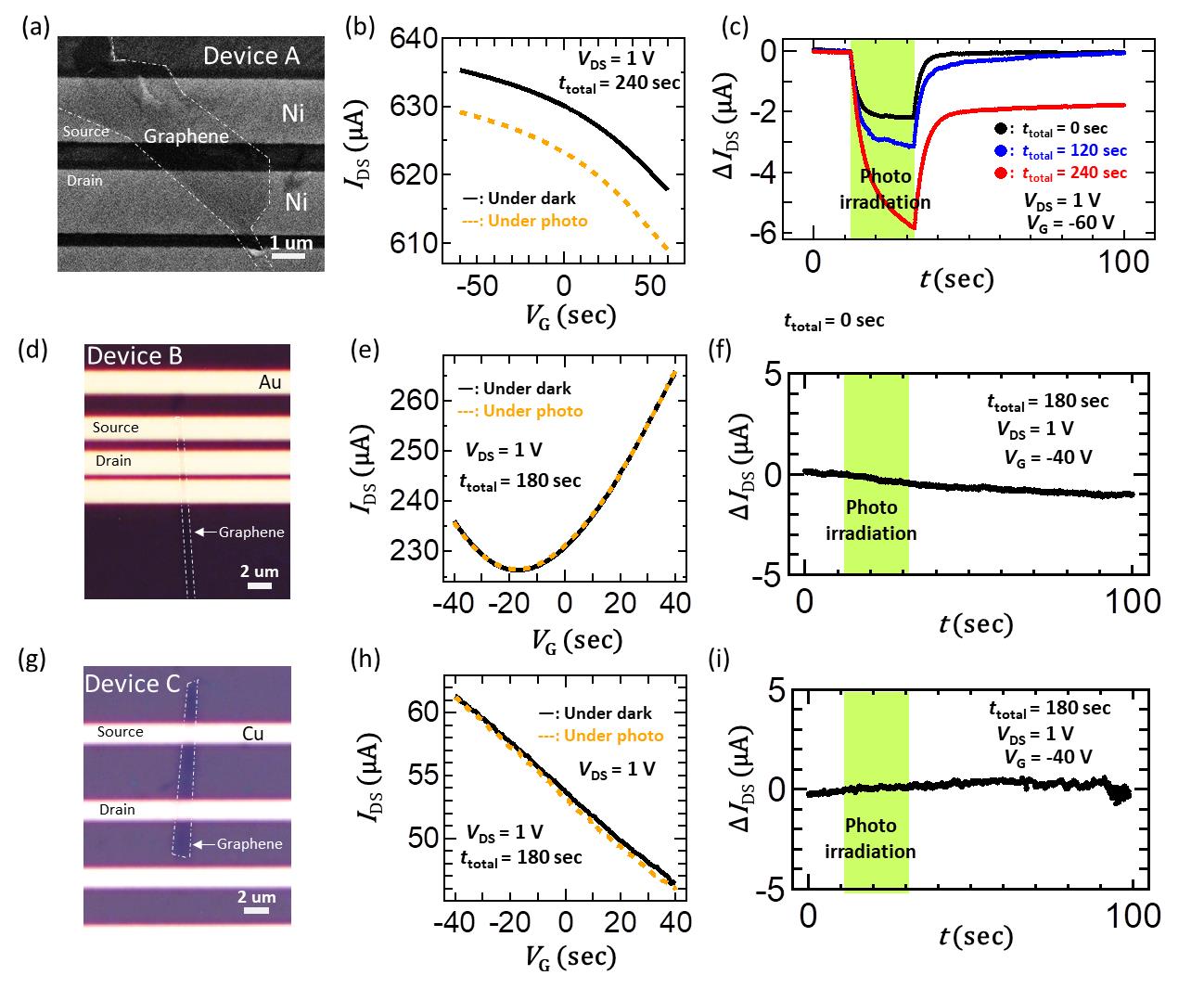


***Figure S11.*** (a)SEM image and (d, g)optical microscope images of suspended graphene devices constructed by Ni, Au and Cu electrodes respectively (these are named device A, B, C respectively). The tri-layer and bi-layer graphene are employed for device A and C, and device B respectively. (b, e, h) *I*_DS_-*V*_G_ curves of O_2_ plasma irradiated device A, B and C (*t*_total_ = 240 sec for device A, 180 sec for device B and C) under dark (black solid line) and photo (orange dot line). (c, f, i) Photoresponse properties of O_2_ plasma irradiated devices (*t*_total_ = 180 sec for device B and C). (c) shows *t*_total_ dependence of photoresponce of device A, the black, blue, red curves are corresponded to *t*_total_ = 0 sec, 120 sec, 240 sec respectively.


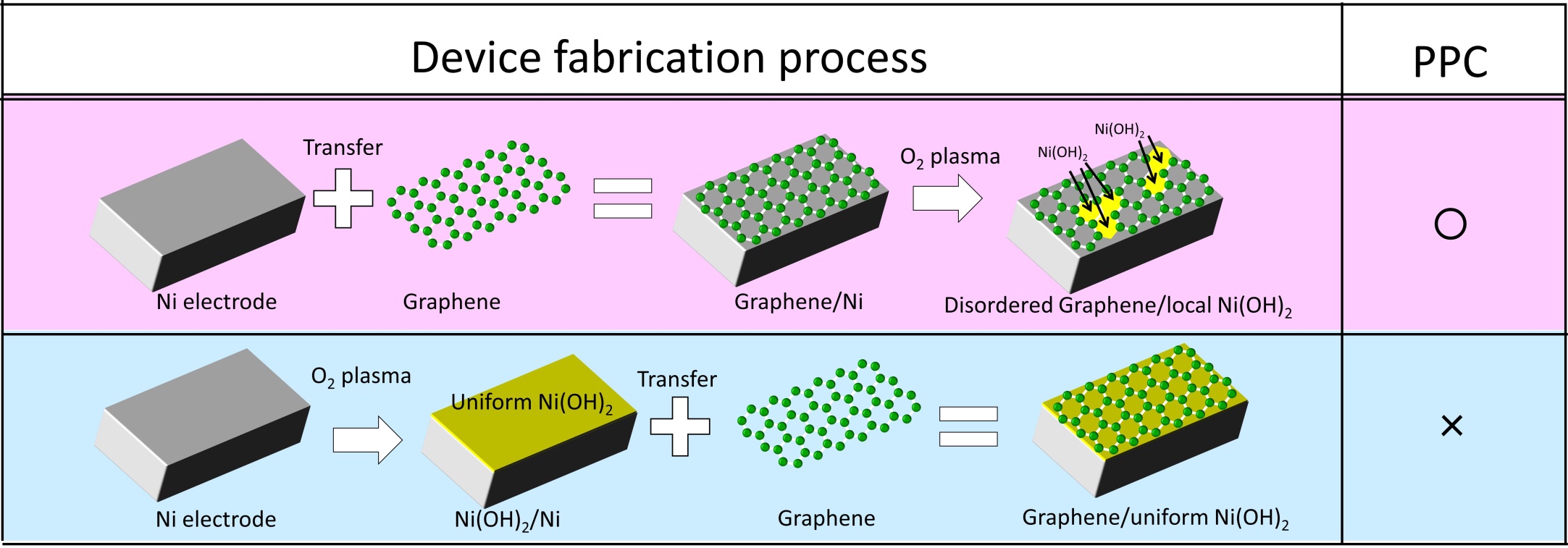


***Figure S12.*** Summary of correlation between device geometry and appearance of PPC.


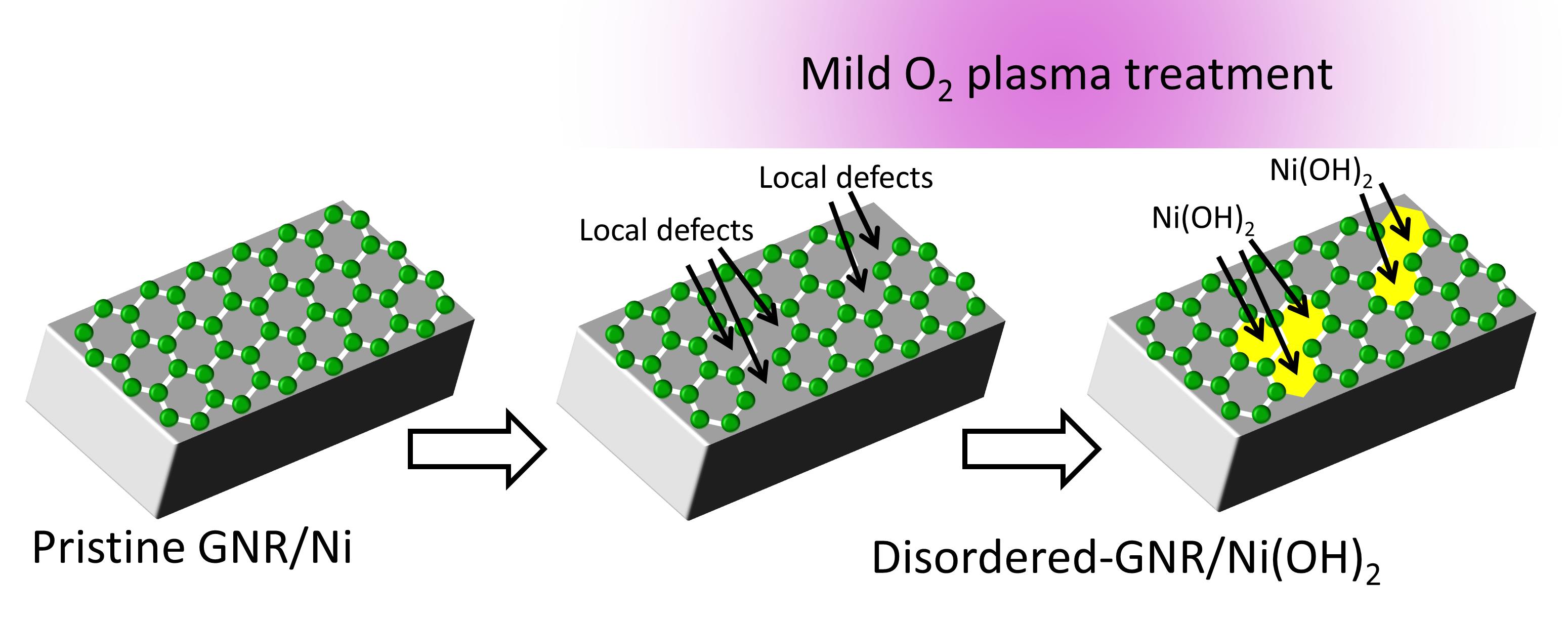


***Figure S13.*** Possible interlayer structures between GNR and Ni where PPC can be observed.


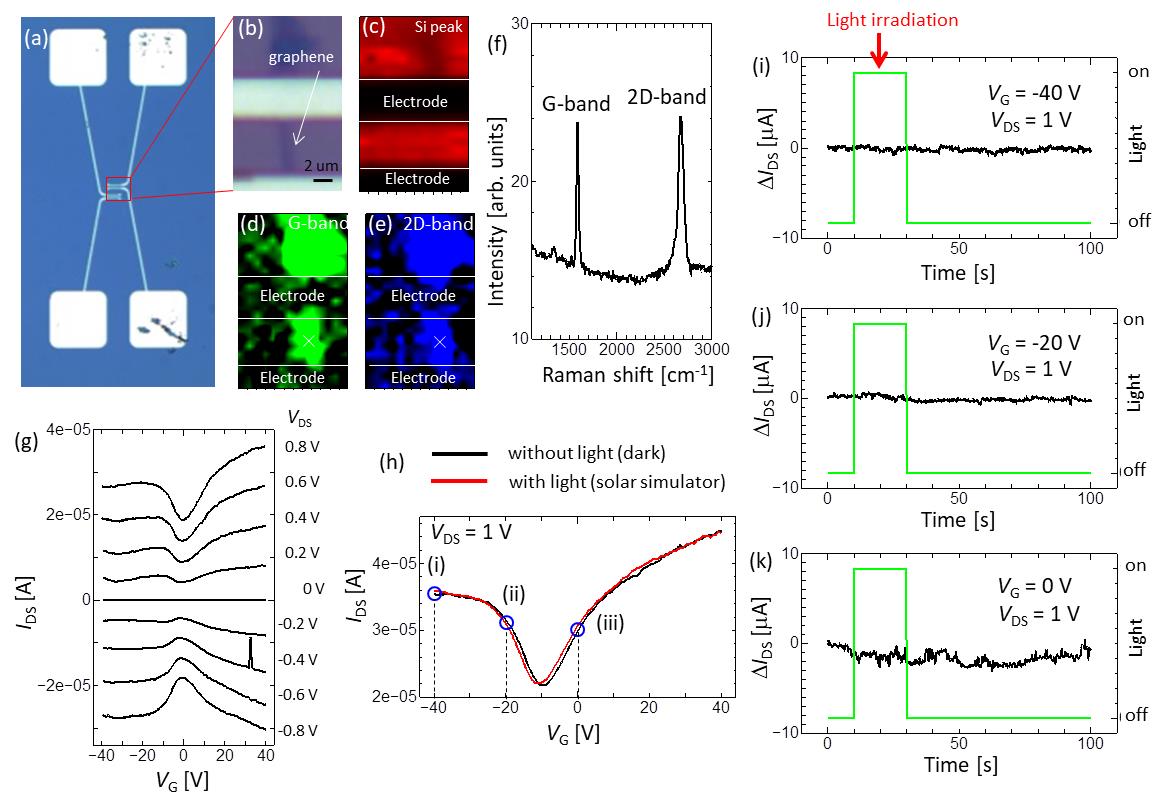


***Figure S14.*** (a,b) Optical microscope and (c-e) Raman mapping (c: Si peak, d: G-band, e: 2D-band) images of supported graphene device. (f) Typical Raman spectrum of supported graphene taken at position × in d. (g) Typical I_DS_-V_G_ curves of supported graphene device measured with various V_DS_. (h) Comparison of I_DS_-V_G_ curve between (red) with and (black) without light irradiation. (i-k) Time profile of ΔI_DS_ measured under different V_G_ conditions ((i) V_G_ = -40 V, (j) V_G_ = -20 V, (k) V_G_ = 0 V, which correspond with the position (i), (ii), and (iii) in h, respectively).
